# Supplementary material for: The Gene vepN Regulated by Global Regulatory Factor veA That Affects Aflatoxin Production, Morphological Development and Pathogenicity in Aspergillus flavus
Source: Toxins (Basel). 2024 Apr 3;16(4):174. doi: 10.3390/toxins16040174 (PMC11054512; doi:10.3390/toxins16040174)
Supplement: Supplementary file 1 [file toxins-16-00174-s001.zip › Supplementary Figure.pdf]

A

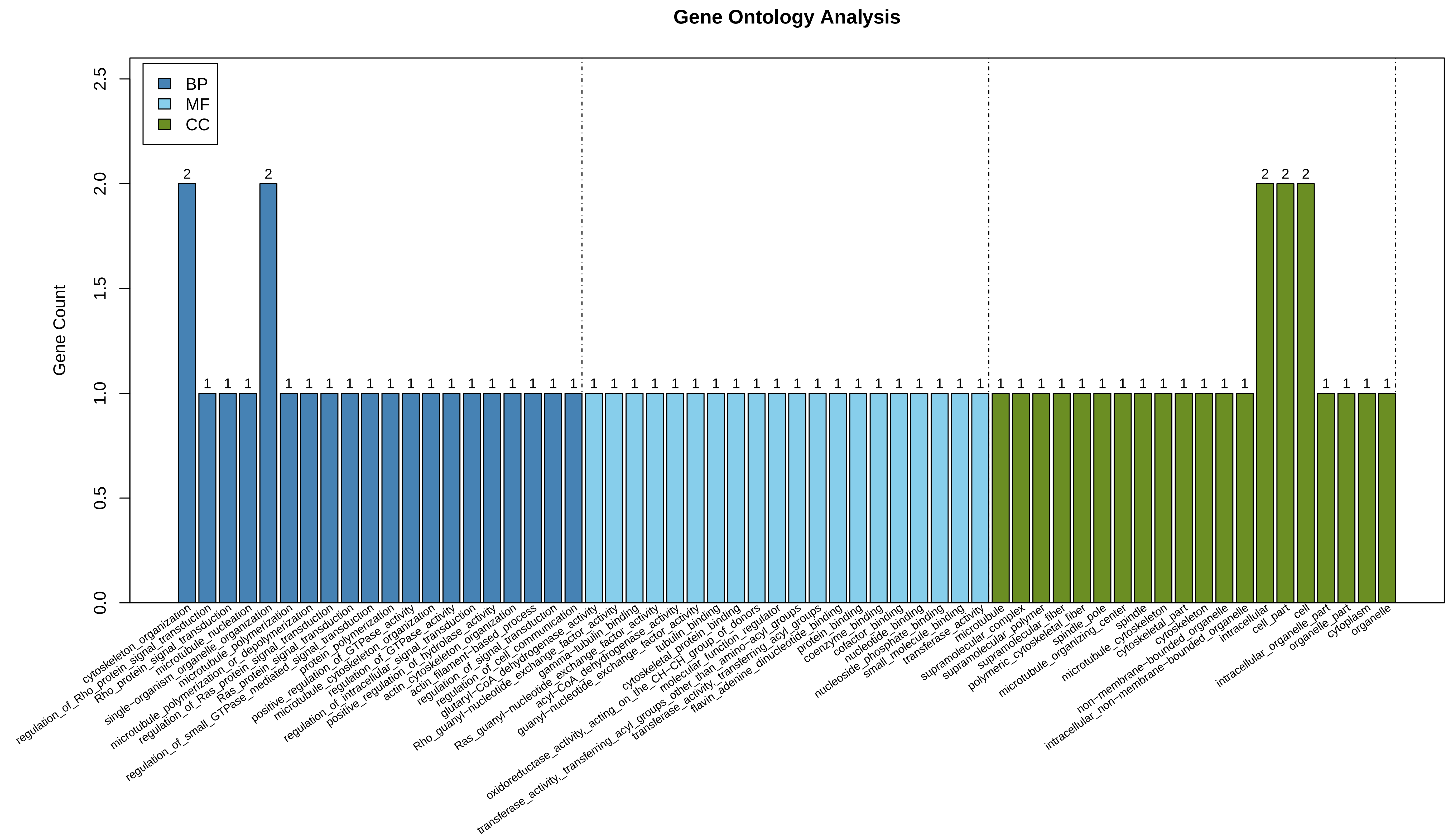
Gene Ontology Analysis
BP
MF
CC
Gene Count
0.0
0.5
1.0
1.5
2.0
2.5
cytoskeleton_organization
regulation_of_Rho_protein_signal_transduction
Rho_protein_signal_transduction
microtubule_nucleation
single−organism_organelle_organization
microtubule_polymerization
microtubule_polymerization_or_depolymerization
regulation_of_Ras_protein_signal_transduction
Ras_protein_signal_transduction
regulation_of_small_GTPase_mediated_signal_transduction
protein_polymerization
positive_regulation_of_GTPase_activity
microtubule_cytoskeleton_organization
regulation_of_GTPase_activity
regulation_of_intracellular_signal_transduction
positive_regulation_of_hydrolase_activity
actin_cytoskeleton_organization
actin_filament−based_process
regulation_of_signal_transduction
regulation_of_cell_communication
glutaryl−CoA_dehydrogenase_activity
Rho_guanyl−nucleotide_exchange_factor_activity
gamma−tubulin_binding
Ras_guanyl−nucleotide_exchange_factor_activity
acyl−CoA_dehydrogenase_activity
guanyl−nucleotide_exchange_factor_activity
tubulin_binding
cytoskeletal_protein_binding
oxidoreductase_activity,_acting_on_the_CH−CH_group_of_donors
molecular_function_regulator
transferase_activity,_transferring_acyl_groups_other_than_amino−acyl_groups
transferase_activity,_transferring_acyl_groups
flavin_adenine_dinucleotide_binding
protein_binding
coenzyme_binding
cofactor_binding
nucleotide_binding
nucleoside_phosphate_binding
small_molecule_binding
transferase_activity
microtubule
supramolecular_complex
supramolecular_polymer
supramolecular_fiber
polymeric_cytoskeletal_fiber
spindle_pole
microtubule_organizing_center
spindle
microtubule_cytoskeleton
cytoskeletal_part
cytoskeleton
non−membrane−bounded_organelle
intracellular_non−membrane−bounded_organelle
intracellular
cell_part
cell
intracellular_organelle_part
organelle_part
cytoplasm
organelle

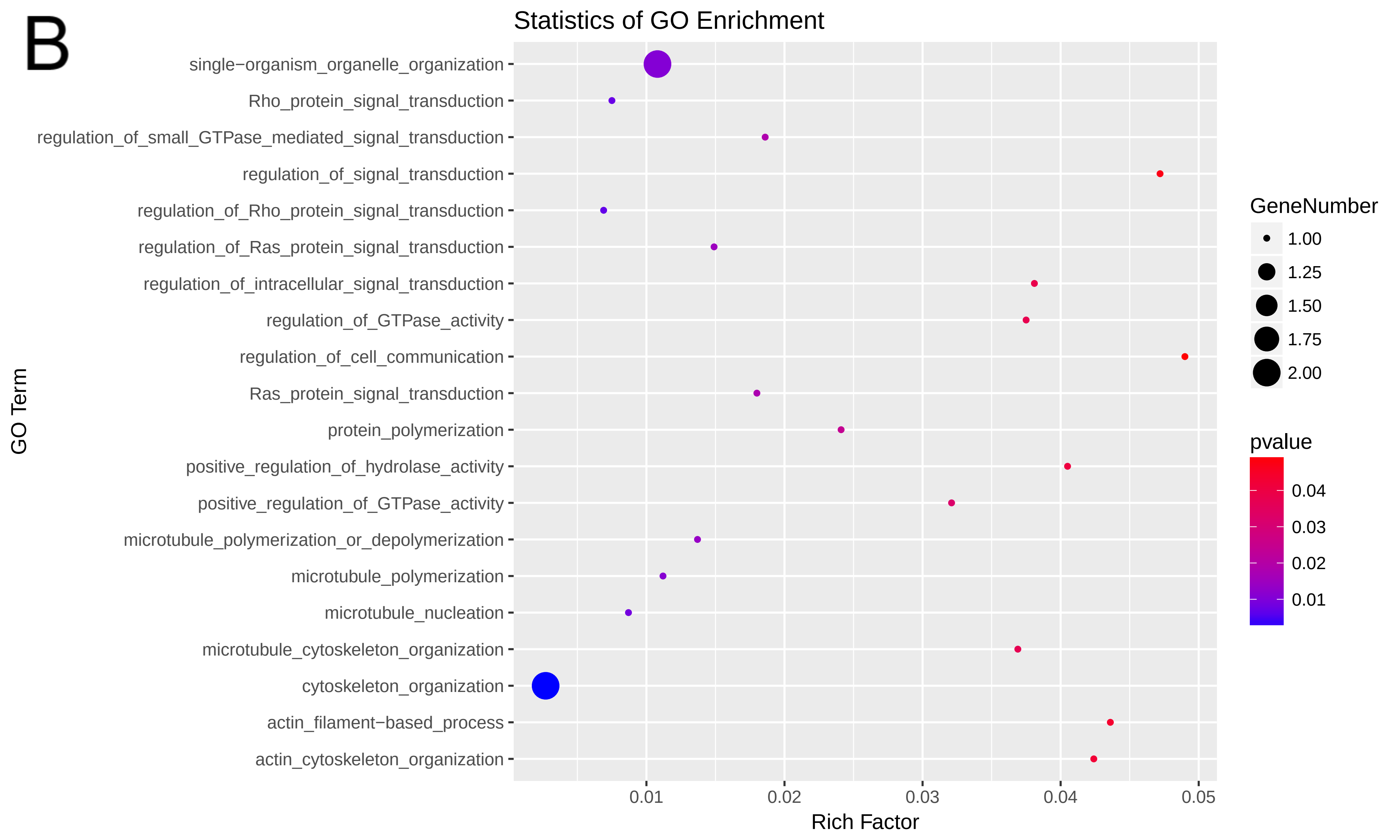
B
Statistics of GO Enrichment
single−organism_organelle_organization
Rho_protein_signal_transduction
regulation_of_small_GTPase_mediated_signal_transduction
regulation_of_signal_transduction
regulation_of_Rho_protein_signal_transduction
regulation_of_Ras_protein_signal_transduction
regulation_of_intracellular_signal_transduction
regulation_of_GTPase_activity
regulation_of_cell_communication
Ras_protein_signal_transduction
protein_polymerization
positive_regulation_of_hydrolase_activity
positive_regulation_of_GTPase_activity
microtubule_polymerization_or_depolymerization
microtubule_polymerization
microtubule_nucleation
microtubule_cytoskeleton_organization
cytoskeleton_organization
actin_filament−based_process
actin_cytoskeleton_organization
GO Term
0.01
0.02
0.03
0.04
0.05
Rich Factor
GeneNumber
1.00
1.25
1.50
1.75
2.00
pvalue
0.04
0.03
0.02
0.01

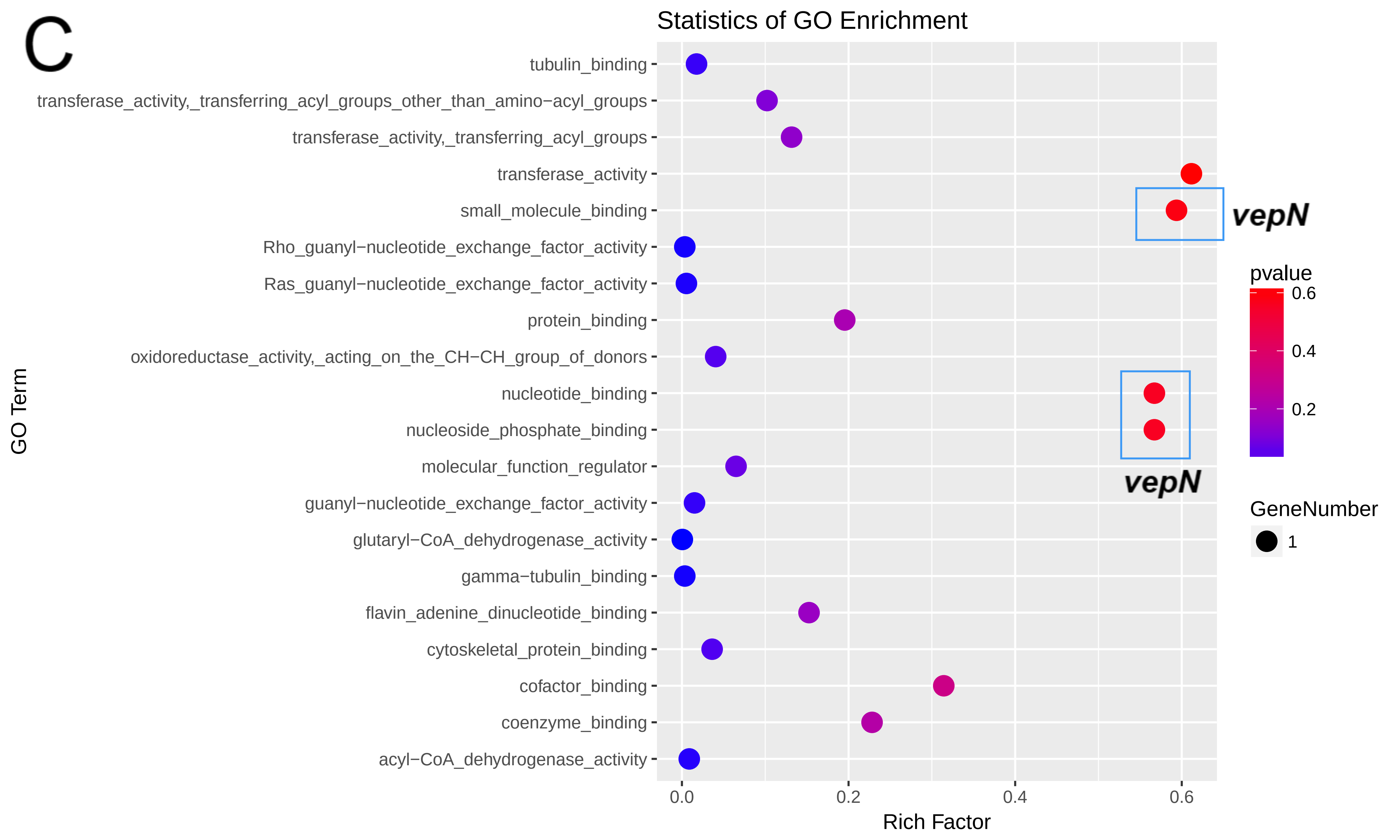
C
Statistics of GO Enrichment
tubulin_binding
transferase_activity,_transferring_acyl_groups_other_than_amino−acyl_groups
transferase_activity,_transferring_acyl_groups
transferase_activity
small_molecule_binding
Rho_guanyl−nucleotide_exchange_factor_activity
Ras_guanyl−nucleotide_exchange_factor_activity
protein_binding
oxidoreductase_activity,_acting_on_the_CH−CH_group_of_donors
nucleotide_binding
nucleoside_phosphate_binding
molecular_function_regulator
guanyl−nucleotide_exchange_factor_activity
glutaryl−CoA_dehydrogenase_activity
gamma−tubulin_binding
flavin_adenine_dinucleotide_binding
cytoskeletal_protein_binding
cofactor_binding
coenzyme_binding
acyl−CoA_dehydrogenase_activity
GO Term
0.0
0.2
0.4
0.6
Rich Factor
vepN
vepN
pvalue
0.6
0.4
0.2
GeneNumber
1

D

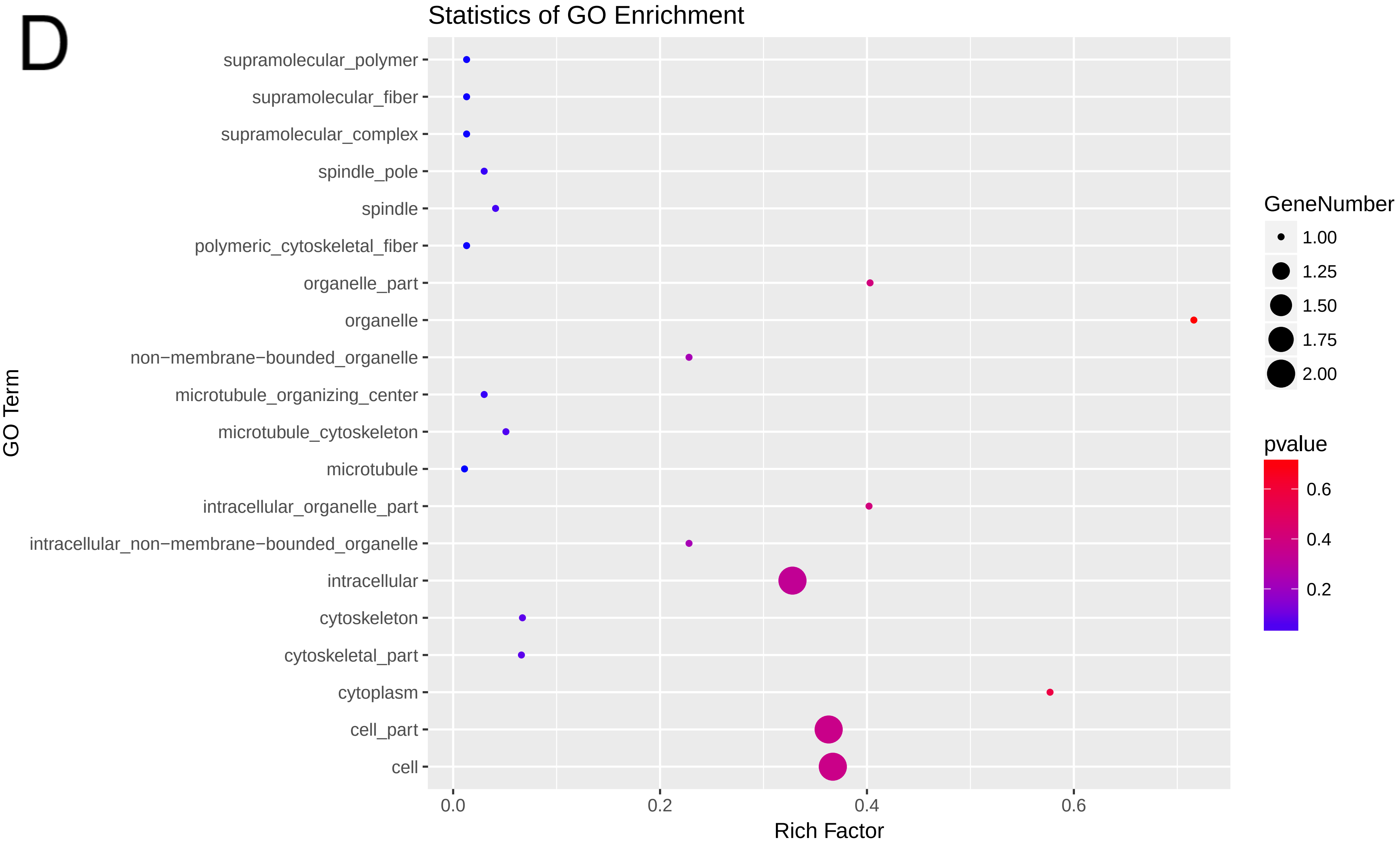
Statistics of GO Enrichment
supramolecular_polymer
supramolecular_fiber
supramolecular_complex
spindle_pole
spindle
polymeric_cytoskeletal_fiber
organelle_part
organelle
non−membrane−bounded_organelle
microtubule_organizing_center
microtubule_cytoskeleton
microtubule
intracellular_organelle_part
intracellular_non−membrane−bounded_organelle
intracellular
cytoskeleton
cytoskeletal_part
cytoplasm
cell_part
cell
GO Term
0.0
0.2
0.4
0.6
Rich Factor
GeneNumber
1.00
1.25
1.50
1.75
2.00
pvalue
0.6
0.4
0.2

Figure S1. GO analysis and statistics of GO enrichment. (**A**) GO analysis. BP: biological process; MF: molecular function; CC: cellular component. (**B**) Statistics of GO enrichment in BP. (**C**) Statistics of GO enrichment in MF. (**D**) Statistics of GO enrichment in CC. The experimental results of the CHIP-seq performed with the VeA antibody are displayed by the results of the GO analysis. The *vepN* gene is enriched in MF, exhibiting binding activity, specifically in nucleotide binding, small molecule binding, nucleoside phosphate binding (It has been marked with a blue border in Figure S1C.)

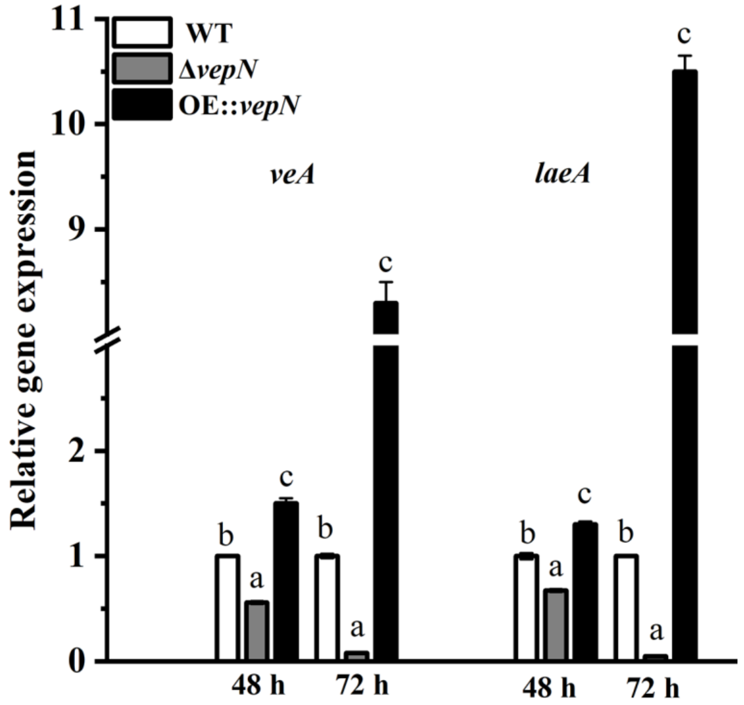

Figure S2 The q-PCR analysis of *veA* and *laeA* genes for conidiation The strains were cultivated on PDA at 30°C for 7 days and then transferred to PDB, cultured for 12 hours. The relative expression was calculated using the method of $2^{-\triangle\triangle Ct}$. The expression of 18S rRNA was used as an internal reference. Values were normalized to the expression levels in the wild-type, considered as one. Error bars represent the standard errors. Error bars represent the standard errors and different letters above the bars represent significantly different values ($p < 0.05$).

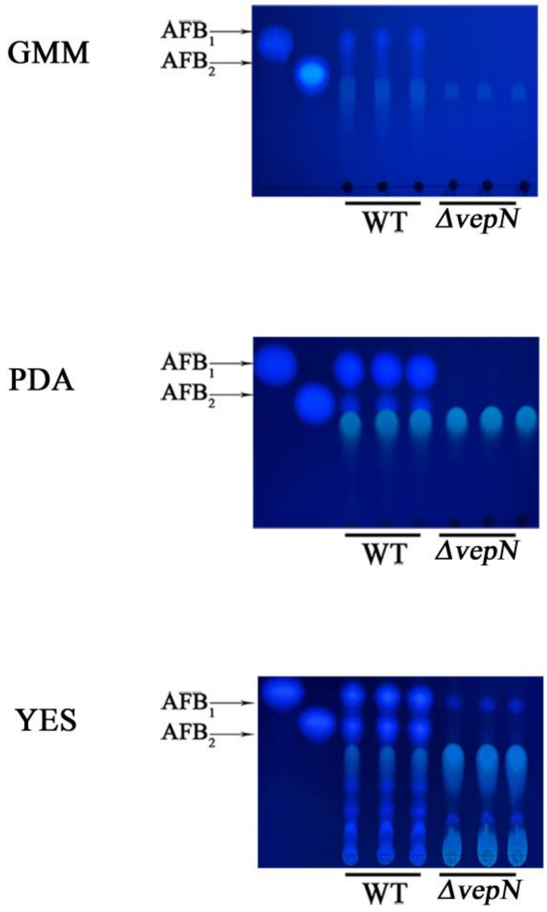

Figure S3 AFB production examined by TLC cultured on GMM, PDA and YES media for 5 days at 30℃.
