# Supplementary material for: The Gene vepN Regulated by Global Regulatory Factor veA That Affects Aflatoxin Production, Morphological Development and Pathogenicity in Aspergillus flavus
Source: Toxins (Basel). 2024 Apr 3;16(4):174. doi: 10.3390/toxins16040174 (PMC11054512; doi:10.3390/toxins16040174)
Supplement: Supplementary file 1 [file toxins-16-00174-s001.zip › Supplementary Tables.pdf]

Table S1. Primers used in strategies and confirmation of the mutant strain.

Primer name	Sequence (5′ -3′)
pyrG-F	GAGAGTTATTCTGTGTCTGACGAAAT
pyrG-R	ATTCTGTCTGAGAGGAGGCA
vepN-5F	CGTGGGGATAATCCCCAAGCCT
vepN-5R	GACACAGAATAACTCTCGCCTCCCTTTTTGCTCTGT
vepN-3F	CTCCTCTCAGACAGAATGGTAGGTAACAGGTGAGGA
vepN-3R	CCGCATCCCTTGTATGGTAGTCAGTAGT
vepN-F	ATGAGACCGCCTGTCCCGGAT
vepN-R	CTCATCTCCGAGAGTACGCGATGA
vepN-5Fp	CGCCACTCAGAGAAACTTCGTG
vepN-5Rp	CTGGCCCAGACCGCATCTGC
vepN-3Fp	GCAAGACTTCCCCTATGGTCCT
vepN-3Rp	CTGACTACCATACAAGGGATGCGG

Table S2. Primers used in strategies and confirmation of the complemented strain.

Primer name	Sequence (5′ -3′)
FA-vector-F	CGAATCATGAAATCAGGGCGCGTCAGC
FA-vector-R	TCTCTGAGTGGCGGCCGCATAGTTAAGCCAGC
FB-5UTR-F	TAACTATGCGGCCGCCACTCAGAGAAACTTCGTG
FB-5UTR-R	GACCTCCACTAGCGCCTCCCTTTTTGCTCTGTTCTG
FC-ble-F	AAAAAGGGAGGCGCTAGTGGAGGTCAACACATCAATG
FC-ble-R	AGGGAGCTTACGCCTAAACAAGTGTACCTGTGCATTCTGG
FD-vepN-F	ACACTTGTTTAGGCGTAAGCTCCCTAATTGGCC
FD-vepN-R	CCTGTTACCTACCGTATTGGGATGAATTTTGTATGCACGCG
FE-3UTR-F	TCATCCCAATACGGTAGGTAACAGGTGAGGAGAACTTATACAAG
FE-3UTR-R	TGACGCGCCCTGATTTCATGATTCGGAAATTCCACTAGACAGAG

Table S3. Primers used in RT-qPCR and sequencing.

Primer name	Sequence (5′ -3′)
18S-F	TGATGACCCGCTCGGCACCTTACGAGAAATCAAAGT
18S-R	GGCCATGCACCACCATCCAAAAGATCAAGAAAGAGC
veA-F	TTCACCGTATTTAGCGCCAAG
veA-R	CATCACGTCGAATCCGCACAC
laeA-F	GAAAGAAAGGTTGCTCGCTGGTA
laeA-F	GTTGAACGCCTCCGACTTGAC
brlA-F	TATCCAGACATTCAAGACGCACAG
brlA-R	GATAATAGAGGGCAAGTTCTCCAAAG
abaA-F	TCTTCGGTTGATGGATGATTTC
abaA-R	CCGTTGGGAGGCTGGGT
nsdC-F	GCCAGACTTGCCAATCAC
nsdC-R	CATCCACCTTGCCCTTTA
nsdD-F	GGACTTGCGGGTCGTGCTA

nsdD-R	AGAACGCTGGGTCTGGTGC
sclR-F	CAATGAGCCTATGGGAGTGG
sclR-R	ATCTTCGCCCGAGTGGTT
aflR-F	AACAAGAGGGCTACCGATGC
aflR-R	TACCATGCCAGCACCTTGAG
aflS-F	ATGTGCGAATCCTATCCCCC
aflS-R	ACGAGGAAACGGAGTGATGG
aflC-F	ACGGAATTTGGTCCCGATGG
aflC-R	GTCAGCATCCAGGTCCGTTC
aflD-F	CGCCTGAGGAGACGGTGTATT
aflD-R	CTGCCTTCAGCGACGGTTAG
aflO-F	CTTTCGGCAGTGACCTAACC
aflO-R	TCTTGAACTATAAGGCGACCA
aflQ-F	GTCGCATATGCCCCGGTCGG
aflQ-R	GGCAACCAGTCGGGTTCCGG
aflM-F	CCGTTTAGATGGCAAAGTGGC
aflM-R	TCACGGGAATGGGCGTAGTT
